# *GmFT4,* a Homolog of *FLOWERING LOCUS T*, Is Positively Regulated by *E1* and Functions as a Flowering Repressor in Soybean

**DOI:** 10.1371/journal.pone.0089030

**Published:** 2014-02-19

**Authors:** Hong Zhai, Shixiang Lü, Shuang Liang, Hongyan Wu, Xingzheng Zhang, Baohui Liu, Fanjiang Kong, Xiaohui Yuan, Jing Li, Zhengjun Xia

**Affiliations:** 1 Key Laboratory of Soybean Molecular Design Breeding, Northeast Institute of Geography and Agroecology, Chinese Academy of Sciences, Harbin, Heilongjiang, China; 2 College of Life Science, Northeast Agricultural University, Harbin, Heilongjiang, China; Ohio State University, United States of America

## Abstract

The major maturity gene *E1* has the most prominent effect on flowering time and photoperiod sensitivity of soybean, but the pathway mediated by *E1* is largely unknown. Here, we found the expression of *GmFT4*, a homolog of *Flowering Locus T,* was strongly up-regulated in transgenic soybean overexpressing *E1*, whereas expression of flowering activators, *GmFT2a* and *GmFT5a*, was suppressed. *GmFT4* expression was strongly up-regulated by long days exhibiting a diurnal rhythm, but down-regulated by short days. Notably, the basal expression level of *GmFT4* was elevated when transferred to continous light, whereas repressed when transferred to continuous dark. *GmFT4* was primarily expressed in fully expanded leaves. Transcript abundance of *GmFT4* was significantly correlated with that of functional *E1,* as well as flowering time phenotype in different cultivars. Overexpression of *GmFT4* delayed the flowering time in transgenic *Arabidopsis*. Taken together, we propose that *GmFT4* acts downstream of *E1* and functions as a flowering repressor, and the balance of two antagonistic factors (*GmFT4* vs *GmFT2a*/*5a*) determines the flowering time of soybean.

## Introduction

The transition from vegetative to reproductive stage is a critical event in the life cycle for seed-propagated plants. Seasonal changes in day length are perceived in leaves, while the responses occur at the apex by long-distance signaling. Florigen, the molecule(s) that migrates from leaves to apical meristem to initiate flowering was proposed by Russian plant physiologist Mikhail Chailakhyan (1937) based on grafting experiments. Recent advances made in *Arabidopsis* (*Arabidopsis thaliana*) and rice (*Oryza sativa*) have demonstrated that FLOWERING LOCUS T (FT) protein produced in leaves, is a florigen that moves through the phloem to the shoot apical meristem (SAM) [1]–[4].

The FT protein, a phosphatidylethanolamine-binding (PEBP)-related kinase, interacts with Flowering Locus D (FD), a bZIP protein, at the vegetative shoot apex. The FT–FD complex subsequently functions to specify flower meristem identity by activating floral meristem genes that start the flowering process, such as *APETALA1*, *FRUITFUL* and *SEPALATA3* [5]–[7].

The expression of *FT* is principally regulated by the *CONSTANS* (*CO*) gene, a central regulator that accelerates flowering in the long day pathway (for long day plants), which is modulated by the circadian clock and day length [8]. The photoperiodic response in *Arabidopsis thaliana* requires the precise regulation of *CO* and *FT* expression coinciding with a photosensitive phase [9]–[10].

Apart from *FT*, two other PEBP family members, *TWIN SISTER OF FT* (*TSF*) and *TERMINAL FLOWER 1* (*TFL1*), are also involved in the control of flowering. *TSF* is a flowering activator, and *TFL1* is a flowering repressor. *TSF* is the closest homolog of *FT* in *Arabidopsis* and is thought to be an additional integrator of flowering time pathways. The mRNA levels of *TSF* and *FT* showed similar patterns of diurnal oscillation and response to photoperiods [11]. Both *FT* and *TSF* are expressed in the vascular tissue of plant leaves but are spatially different, with *TSF* expressed mainly in hypocotyls while *FT* expressed in cotyledons and leaves [11], [12]. *TFL*1, a shoot meristem identity gene, is expressed specifically in the shoot apical meristem (SAM) and represses the transition to flowering [13]–[16].

FT acting as a floral activator is widely conserved in plant species, although *FT* mRNA can be regulated by distinct mechanisms among different species even within long-day (LD) or short-day (SD) plant species [17]. Overexpression of *FT* orthologs, *Hd3a* and *RFT1,* generally showed an early-flowering phenotype, while mutations in *FT* led to a late flowering phenotype in rice [18]–[20]. Similarly, many functional *FT* orthologs were characterized, e.g. *GmFT2a* and *GmFT5a* in soybean (*Glycine max*) [21]–[22], *ZCN8* in maize (*Zea Mays*) [23]–[24], *SFT* in tomato (*Solanum lycopersicum*) [25], *TaFT* in wheat (*Triticum aestivum*) [26], *HvFT* in barley (*Hordeum vulgare*) [27], *PnFT1/2* in Pharbitis (*Pharbitis nil*) [28], *HaFT1* and *HaFT4* in sunflower (*Helianthus annuus*) [29]. In sugar beet, two *FT* orthologs *BvFT1* and *BvFT2* act antagonistically. *BvFT2* is functionally conserved with *FTs* from other plant*s* and is essential to activate flowering. In contrast, *BvFT1* represses flowering and is crucial for the vernalization response in sugar beets [30].

It is generally accepted that the clock-controlled *CO*-*FT* external coincidence mechanism is conserved in higher plants. However, each plant species has evolved its own unique mechanisms to induce flowering under optimal conditions. In rice, *Hd1*, an ortholog of *CO*, promotes flowering under SD conditions, while another rice *CO*-like gene, *Ghd7*, acts as a floral repressor under LD conditions and suppresses the transcription of *Ehd1*, a floral activator of multiple flowering signals [20], [31]–[32]. In barley, *HvCO9*, an ortholog of *CO*, acts as a negative regulator of flowering under non-inductive SD conditions. In Pharbitis, *PnFT* mRNA abundance was not related to *PnCO* expression [28]. It was reported *Lotus japonicas* (a model legume) might lack the upstream positive regulator CO [33]. In pea (*Pisum sativum*), *COLa* is the most homologous gene, but is not the ortholog to *AtCO* in terms of the function. The diurnal expression rhythm of *COLa* under long days is more similar to *Arabidopsis COL1* and *COL2* [34], which have little effect on flowering time [35], and the expression of *COLa* is not obviously altered in *late1* mutants (*LATE1* is an ortholog of *Arabidopsis GIGANTEA*) [34], while in *Arabidopsis AtCO* expression is constantly dampened in *gi-2* mutants [36]. In soybean, the maturity gene *E1*, which has the most prominent effect on flowering time and photoperiod sensitivity, is a legume-specific gene [37]. Hence, it is speculated that there might be significant differences in the mechanisms of flowering time regulation between legume and the model species *Arabidopsis/*rice.

“Photoperiodism ” in soybean was discovered in 1920, but the molecular mechanism is poorly understood. Soybean is typically a short-day (SD) photoperiod-sensitive plant: flowering is induced when the daylength becomes shorter than a critical length. Each soybean cultivar is generally restricted to a very narrow range of latitudes due to photoperiod sensitivity. Flowering time and maturity in soybean are important quantitative traits that contribute to photoperiod adaptability, domestication, and productivity.

To date, eight flowering time or maturity loci, designated *E1* to *E8* [38]–[45], along with the *J* locus for the long juvenile period trait, with which soybean flowers late even under short days (SDs) [46], have been characterized genetically. Of these, *E1*, *E3*, and *E4* are involved in photoperiod responses [40]–[41], [44], [47]–[49]. *E3* and *E4* encode homologs of the photoreceptor phytochrome A (PHYA) [50]–[52]. *E2* encodes a homolog of GIGANTEA (GI) [53], a key regulator of photoperiodic flowering in *Arabidopsis* that functions upstream of *CO* and *FT* [36], [54]. *E1* encodes a legume-specific protein, which contains a putative bipartite nuclear localization signal, a region distantly related to DNA-binding B3 domain and a helix–turn–helix structure, and might function as a transcription factor [37]. In addition, two of the *FT* homologs, *GmFT2a* and *GmFT5a* are responsible for inducing flowering under short-day conditions [22]–[23]. *GmFT2a* and *GmFT5a* are regulated by PHYA: Functional *E3* and *E4* genotypes suppressed the expression of *GmFT2a* and *GmFT5a* under long-day conditions and delayed flowering, whereas double-recessive *e3e4* genotypes induced *GmFT2a* and *GmFT5a* expression and promoted early flowering regardless of day length [22]–[23].

In our previous study, we proposed that *E1* is a part of the phytochrome A signaling pathway and antagonistically determines the expression level of *GmFT2a* and *GmFT5a* [37]. Long-days (LDs) are necessary for the induction of *E1* expression, whereas loss-of-function alleles at *E3* or *E4* can result in some degree of suppression of the *E1* transcription and correspondingly elevated *GmFT2a* and *GmFT5a* expression. When a functional *E1* gene was transformed into the early-flowering cultivar Kariyutaka, transgenic plants overexpressing *E1* displayed late flowering and suppression of *GmFT2a* and *GmFT5a* transcript levels, indicating that the transcript level of *E1* was negatively correlated with that of *GmFT2a* and *GmFT5a,* but positively with flowering time.

In this study, we found the transcript level of *FT* ortholog *GmFT4* (*Glyma08g47810*) in soybean was strongly up-regulated in transgenic soybean overexpressing *E1,* and is tightly associated with *E1* or *e1-as* expression in soybean cultivars. Ectopic expression analysis in *Arabidopsis* demonstrated that *GmFT4* acts as a flowering repressor. The diurnal rhythm and tissue-organ expression pattern of *GmFT4* were also analyzed. Taken together, we propose that *GmFT4* is a key regulator in the *E1* mediated photoperiodic flowering pathway, and soybean has developed its unique pathway to control flowering through coordinated regulation between the flowering promoters *GmFT2a/GmFT5a* and repressor *GmFT4*.

## Materials and Methods

### Plant Materials and Growth Conditions

Soybean cultivars Kariyutaka, HeiNong48, Mufeng7, HN112, HN89, HX3, *E1* near-isogenic line Harosoy-*E1* and Harosoy-*e1*, Jack, Jinlin35, Sidou11, Yanhuang3 and Sakamotowase were used. Kariyutaka, HeiNong48, Mufeng7 and Sakamotowase are photoperiod-insensitive cultivars and flower early under both SDs and LDs. HN112, HN89, HX3, Jack, Jinlin35, Sidou11and Yanhuang3 are photoperiod-sensitive cultivars and flower late under LDs. HX3 exhibits the long juvenile period trait, and flowers late even under SDs. Harosoy-*E1* and Harosoy-*e1* are *E1* near-isogenic lines. Harosoy-*E1* carrying the dominant functional *E1* allele is a late flowering phenotype. Harosoy-*e1* carrying the recessive *e1* allele, with a single missense point mutation, demonstrates an early flowering phenotype. Plants were grown in an artificial climate chamber under either SDs (12 h:12 h light/dark) or LDs (16∶8 h light/dark) at 28°C under a light fluency of 200–300 µmol m^−2^ S^−1^. On the 16th day after emergence, fully expanded trifoliolate leaves were sampled 4 h after dawn from three individual plants for real-time PCR analysis.

For diurnal rhythmic expression analysis, soybean cultivar Harosoy-*E1* was used. Soybean plants were kept under SDs (12 h of light) and LDs (18 h of light) for 16 days before being transferred into continuous light or dark conditions. Pieces of fully expanded trifoliolate leaves from three individual plants were sampled every 2 h starting at dawn under SD, LD and continuous light conditions, and sampled every 4 h under continuous dark conditions for real-time PCR analysis.

For tissue-organ analysis, soybean cultivars Kariyutaka, transgenic soybean overexpressing *E1* in Kariyutaka, Harosoy-*E1*, Harosoy-*e1* and HX3 under LDs were used. Three sets of unifoliolate leaves, unexpanded and fully expanded trifoliolate leaves, apical meristems, petioles, stems and flowers from three individual plants were sampled for real-time PCR analysis.

### RNA Isolation, cDNA Synthesis and Quantitative Real-time PCR Analysis

Total RNA from leaves, apical meristems, petioles and stems was extracted using TRIzol (Invitrogen, Carlsbad, CA, USA) method and total RNA from flowers was extracted using TransZol plant (TransGen, Beijing, China) according to the manufacturer's instructions. The RNA was treated with RNase-free recombinant DNase I (Takara, Dalian, China). The integrity of the RNA was checked electrophoretically and quality assessment of total RNA was checked with NanoDrop™ ND-2000 c Spectrophotometer (Thermo Scientific, Wilmington, DE, USA). The isolated RNA was then subjected to reverse transcription using the SuperScript™ III Reverse Transcriptase kit.

Quantitative real-time PCR was performed on each cDNA sample with the SYBR Green Master Mix (TransStart Top Green qPCR SuperMix, Beijing, China) on chromo4 real-time PCR detection system (Bio-Rad, USA) according to the manufacturer’s protocol. The measured Ct values were converted to relative copy-numbers using the ΔΔCt method. Amplification of *TUA5* was used as an internal control to normalize all data. Primers used were *TUA5*(Glyme05g29000.1)-F 5′-TGCCACCATCAAGACTAAGAGG-3′ and *TUA5*-R 5′- ACCACCAGGAACAACAGAAGG-3′; *GmFT4*-F 5′–TTGGATCCCTTCACGAGTTC -3′ and *GmFT4*-R 5′- TCCCTAGGTCATTTCCACGA -3′; *GmFT2a*-F 5′–ATCCCGATGCACCTAGCCCA -3′ and *GmFT2a*-R 5′- ACACCAAACGATGAATCCCCA -3′; *GmFT5a*-F 5′- AGCCCGAACCCTTCAGTAGGGA -3′; *GmFT5a*-R 5′- GGTGATGACAGTGTCTCTGCCCA -3′; *E1*-F 5′- CACTCAAATTAAGCCCTTTCA -3′; *E1*-R 5′- TTCATCTCCTCTTCATTTTTGTTG -3′;To enable statistical analysis, three fully independent biological replicates were obtained and subjected to real-time PCR run in triplicate. Raw data were standardized as described previously [55].

### Sequence Alignment and Phylogenetic Analysis

Protein sequences of *GmFT4* and its homologs were obtained from NCBI or Phytozome and were aligned using Clustal X2 (protein weight matrix using the Gonnet Series with a gap penalty of 10.00, a gap length penalty of 0.20, and a delay-divergent cutoff of 30%), and phylogenetic analysis was performed by using MEGA4 with the UPGMA method and 500 bootstrap iterations.

### Ectopic Expression of *GmFT4* in *Arabidopsis*

The coding region sequence of *GmFT4* from *E1* overexpression transgenic lines was first cloned into the pGEM®-T Easy vector (Promega, Madison, WI, USA) with the primer pair (5′-CTATATCAATGGACCCCCTTGTTC-3′) and (5′-AAGAAGGGTCTTCATCTCCTTCG-3′). *GmFT4* coding region was then PCR amplified from pGEM®-T-*GmFT4* vector with primers pair (5′-**GGCTTAAU**AATGGACCCCCTTGTTCTT-3′) and (5′-**GGTTTAAU**GGTCTTCATCTCCTTCGTCC-3′), which contained a tail of 8 nt (marked as **Bold fonts**) in addition to the sequence specific to the target DNA fragment. The sequence was inserted into the pCAMBIA230035Su vector with the USER™ cloning technique [56], driven by the cauliflower mosaic virus 35 S promoter, with *NptII* as the selectable marker. *Arabidopsis* Col-0 plants were transformed by the floral dip method [57]. Transformants were selected on 1/2 MS medium containing 50 mg/L kanamycin. Seeds from each T_1_ plant were individually collected. Selected T_2_ plants were propagated, and homozygous overexpression lines were confirmed by semi-quantitative RT-PCR analysis using a gene specific primer pair of 5′- ATGGACCCCCTTGTTCTTGGAC -3′ and 5′- TCATCTCCTTCGTCCACCCCA -3′.

### Flowering Time Measurements of Transgenic *Arabidopsis*

*Arabidopsis* plants were grown in soil in an artificial climate chamber under long-day conditions (16∶8 h light/dark) at 22°C to 24°C with 60% relative humidity. Flowering time was recorded when the floral bolt was 1 cm high, meanwhile the total number of rosette leaves were counted. About ninety plants were measured and subjected to statistical analysis.

## Results

### *GmFT4* Expression was Up-regulated in Transgenic Soybean Overexpressing *E1*

**Figure 1:**
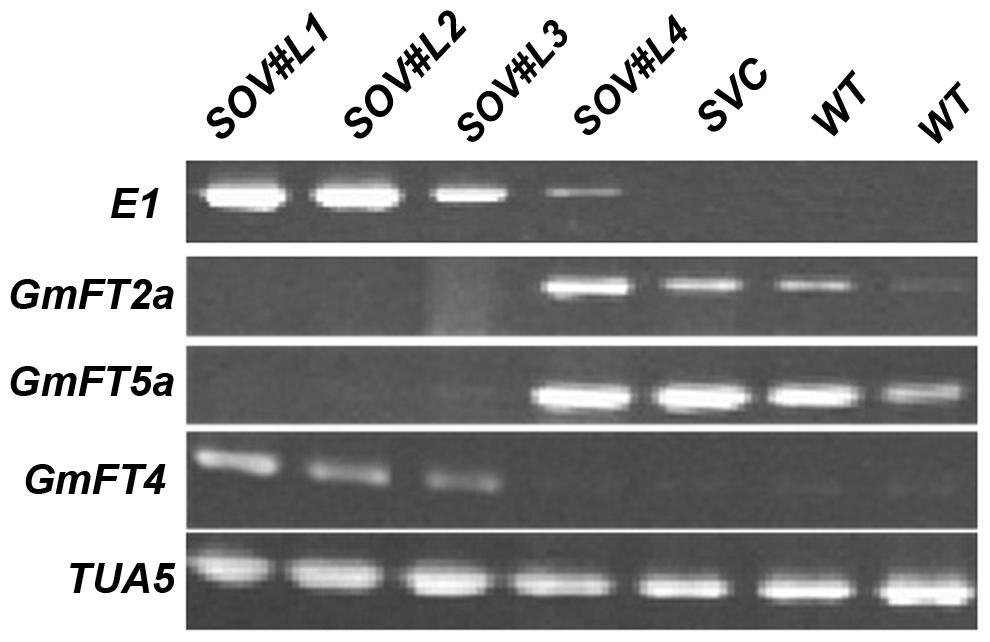
Expression analysis of *FT*-like genes in transgenic soybean overexpressing *E1* and WT plants under LDs. Fully expanded trifoliolate leaves were sampled 4-quantitative RT-PCR. SOV#L1, SOV#L2 and SOV#L3 were T_2_ transgenic plants from transgenic T_0_ line TG4, that has three copy exogenous *E1* insertions. SOV#L4 was T_2_ transgenic plant from transgenic T_0_ line TG2, that has 7–8 copy exogenous *E1* insertions [37]. SVC, transformation vector only (i.e., vector control); WT, Kariyutaka. The *TUA5* gene was used as a control.

In our previous study, when *E1* was overexpressed in soybean cultivar Kariyutaka, expression levels of *GmFT2a* and *GmFT5a* were decreased in the transgenic soybean compared with the wild-type [37]. We further investigated expression of other *FT* homologs in transgenic and wild-type soybean, and found that the expression of *GmFT4*, a homolog of *FT*, was increased in transgenic soybean lines SOV#L1, SOV#L2 and SOV#L3 compared with that of SVC(transformed vector only) and wild-type (Figure 1). Since *GmFT4* showed an expression pattern oppsite to *GmFT2a* and *GmFT5a*, further analysis of *GmFT4* was performed in order to understand the functional role of *GmFT4* in controlling flowering time.

### GmFT4 was Grouped within the FT-like Clade and Carries Functionally Important FT Signatures

**Figure 2:**
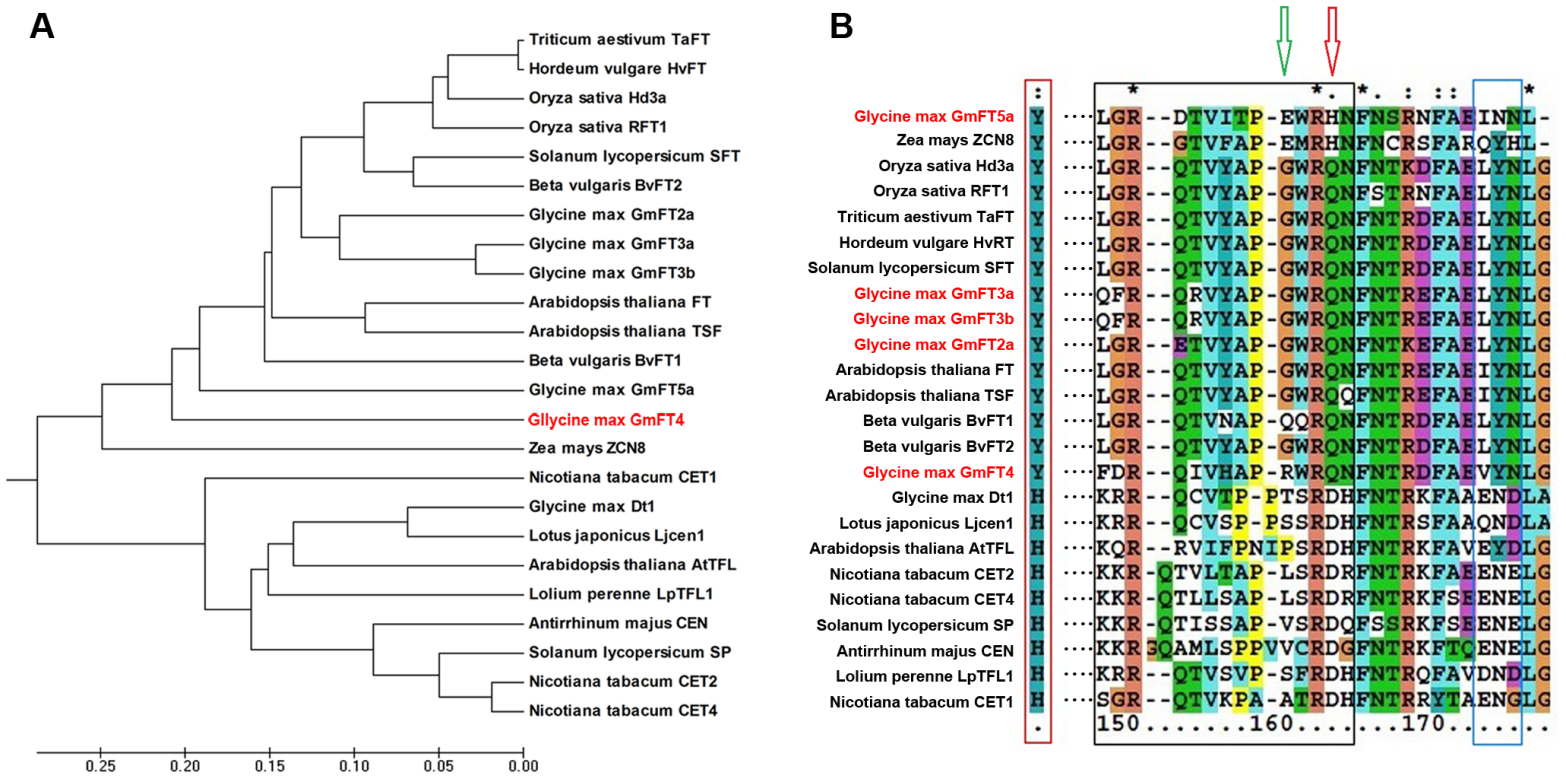
Sequence comparison of FT/TFL1 family members from flowering plants. (**A**) Alignment of sequences of FT/TFL1 family members from flowering plants. The Tyr85/His88 residue, that lies at the entrance to the ligand-binding pocket, distinguishing all FT from TFL1 members is boxed in red. Segment B is boxed in black: the Asp144/Gln140 residue distinguishing all FT from TFL1 members is indicated by red arrow. The predicted key residue, which may play an important role in functional diversification is indicated by green arrow. (**B**) Phylogenetic tree of GmFT4 and other FT/TFL1 family members, most of which have been functionally characterized.

FT/TFL1 family members that have been functionally characterized from a wide range of monocotyledonous and dicotyledonous plant species were collected and subjected to phylogenetic analysis (Figure 2A). The results indicated that GmFT4 was grouped into the FT-like clade. Sequence alignment was also conducted (Figure 2B). Tyr85(Y) in FT and the corresponding His88 (H) in TFL1 that lie at the entrance to the ligand-binding pocket have been identified to be important for the functional diversification between FT and TFL1 [58]. Also, a 14-amino-acid external loop and a 3-amino-acid triad have also been reported to be critical for FT/TFL function determination [59]. This 14-amino-acid segment and triad segment evolves very rapidly in TFL1 orthologs, but kept almost unchanged in FT orthologs. The key residue at 140 which lies in the external loop segment unambiguously distinguishes FT [Gln140 (Q)] from TFL1 homologs [Asp144(D)]. As shown in Figure 2B, the functionally determinant residues for the FT clade in the GmFT4 protein are Tyr81(Y) and Gln146(Q). When compared with that of other FT proteins, the VYN triad is relatively invariable, however, the 14-amino-acid external loop in GmFT4 protein is more variable.

### *GmFT4* Expression was Generally Elevated under LDs and was Associated with Flowering Time

**Figure 3:**
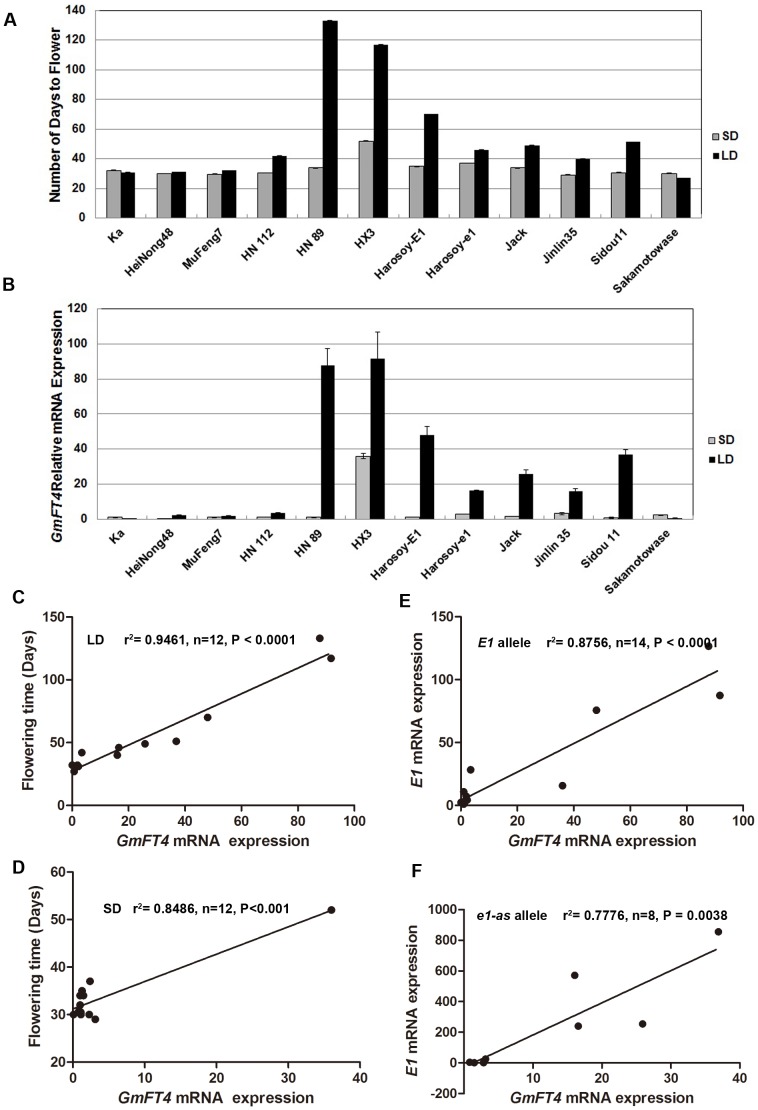
Expression analysis of *GmFT4* in different soybean cultivars under SDs and LDs by real-time RT-PCR. (**A**) Number of days to flowering. (**B**) Evaluation of *GmFT4* transcript levels in fully expanded trifoliolate leaves by real-time RT-PCR. Transcript levels relative to *TUA5* were represented in each treatment; Soybean cultivar Kariyutaka under SDs was used as control. Values represent means of three biological replicates; error bars indicate standard deviation.(**C and D**) Correlation analysis between *GmFT4* mRNA and flowering time of different soybean cultivars under LDs and SDs. Results showed that *GmFT4* mRNA expression was significantly correlated with flowering time of different soybean cultivars under both SDs and LDs. (**E and F**) Correlation analysis between *GmFT4* mRNA expression and *E1* mRNA expression in cultivars carrying *E1* allele and cultivars carrying *e1-as* allele. *GmFT4* expression is significantly correlated with the *E1* expression in cultivars carrying both *E1* allele and cultivars carrying *e1*-as allele.

In order to assess whether the expression of *GmFT4* is involved in the photoperiod pathway, transcript levels of *GmFT4* in different soybean cultivars under both LDs and SDs were investigated (Figure 3A, B). Generally, *GmFT4* was highly induced under LDs, and repressed under SDs in most soybean cultivars, especially in photoperiod-sensitive cultivars, e.g. HN112, HN89, HX3, Harosoy-*E1*, Harosoy-*e1,* Jack, Jilin 35 and Sidou 11. Whereas, in photoperiod-insensitive cultivars, such as Kariyutaka, Heinong 48, Mufeng 7 and Sakamotowase, *GmFT4* expression level was very low under both SDs and LDs. Relatively higher *GmFT4* expression levels under SDs than under LDs were observed in Kariyutaka and Sakamotowase, however, it may not be meaningful to compare them since both were at very low levels. *E1*, *E3*, and *E4* were reported to be involved in photoperiod responses. Kariyutaka has the *e3e4* genotype (double recessive *E3*, *E4*) and showed a suppressed *E1* expression. Both Heinong 48 and Mufeng 7 carry the *e3* genotype (*E3* recessive). Sakamotowase carries the *e1-fs* genotype (a frame shift mutation of *E1*). Therefore, we might be able to hypothesize *E3*, *E4* and *E1* regulate the photoperiod response of soybean via *GmFT4*.

Meanwhile, we found *GmFT4* expression was significantly correlated with flowering time. Late flowering soybean cultivars were displaying high levels of *GmFT4* expression, while early flowering cultivars showed opposite trends (Figure 3B). Even under SDs, long juvenile cultivar HX3 that exhibited delayed flowering under SDs also showed a relatively high *GmFT4* expression. We then conducted correlation analysis (Figure 3C, D), where *GmFT4* expression was significantly correlated with flowering time under both LDs (Figure 3C) (r^2^ = 0.9461***, n = 12, P<0.0001) and SDs (Figure 3D) (r^2^ = 0.8486**, n = 12, P<0.001), suggesting that *GmFT4* might act as flowering repressor in soybean.

### *GmFT4* Expression is Associated with *E1* Expression and *E1* Genotype

To evaluate the functional consequence between *GmFT4* and *E1*, *E1* expression and *E1* allelic variations were also investigated. As shown in Table 1, cultivars Kariyutaka, Heinong 48, Mufeng 7, HN112, HN89, HX3 and Harosoy-*E1* carry the *E1* genotype. Cultivars Harosoy-*e1*, Jack, Jilin 35 and Sidou 11 carry the *e1-as* genotype. Allele *e1-as* that harbors a 1-bp mutation, is a leaky allele and may retain partial *E1* function. The *e1-fs* allele in cultivar Sakamotowase, has a 1-bp deletion, resulting in a premature stop codon, and is nonfunctional [37]. *GmFT4* transcript level in Harosoy-*E1* was higher than that in Harosoy-*e1* under LDs (Table 1). Among cultivars carrying the *E1* allele, expression level of *GmFT4* fluctuated with the *E1* expression level (Table 1). Apparently, higher *GmFT4* expression level occurred in plants or cultivars with high expression level of *E1*, while low expression level of *GmFT4* was coupled with lower *E1* expression. Similar trends were also observed in cultivars carrying *e1-as* allele, however, much higher *e1-as* expression compared to *E1* expression was associated with the equivalent amount of *GmFT4* transcripts, possible due to the dosage effect since *e1-as* is less functional compared to *E1*. Statistical analysis showed that *GmFT4* expression was significantly correlated with the *E1* expression in cultivars carrying the *E1* allele (r^2^ = 0.8756***, n = 14, P<0.0001) and in cultivars carrying the *e1*-as allele (r^2^ = 0.7776*, n = 8, P<0.01) (Figure 3E and F). In cultivar Sakamotowase with the *e1-fs* genotype, the expression of *GmFT4* was at very low level. These results indicated *GmFT4* expression level is dependent on the amount of functional *E1* transcripts.

**Table 1:** *GmFT4* expression is associated with flowering time, *E1* genotype and *E1* expression.

Cultivar or NIL	Flowering time average±s.d.(days)		*GmFT4* relative expression average±s.d.		*E1* genotype	*E1* relative expression average±s.d.	
	SD	LD	SD	LD		SD	LD
Kariyutaka	32±0.25	30±0.11	1.00±0.11	0.10±0.01	*E1*	1.00±0.17	2.33±0.25
Heinong 48	30±0.15	31±0.13	0.10±0.01	2.26±0.33	*E1*	2.33±0.22	4.26±0.33
MuFeng 7	30±0.25	32±0.09	1.12±0.15	1.94±0.24	*E1*	1.57±0.15	7.16±0.82
HN112	31±0.15	42±0.11	1.10±0.12	3.48±0.24	*E1*	2.98±0.12	28.3±0.93
HN89	34±0.25	133±0.13	1.02±0.31	87.73±9.53	*E1*	10.78±1.32	126.53±6.57
HX3	52±0.15	117±0.09	36.00±1.41	91.77±14.83	*E1*	15.73±1.43	87.42±4.83
Harosoy-*E1*	35±0.28	70±0.11	1.27±0.04	48.01±5.09	*E1*	1.83±0.11	75.7±7.46
Harosoy-*e1*	37±0.16	46±0.13	2.83±0.12	16.56±0.15	*e1-as*	1.97±0.13	240.83±8.55
Jack	34±0.25	49±0.09	1.47±0.04	25.89±2.33	*e1-as*	1.27±0.14	254.22±13.41
Jilin 35	29±0.26	40±0.21	3.12±0.45	16.06±1.44	*e1-as*	25.15±2.45	571.78±25.44
Sidou 11	31±0.25	51±0.11	0.82±0.31	36.91±2.75	*e1-as*	4.84±1.31	855.05±19.74
Sakamotowase	30±0.25	27±0.13	2.27±0.040	0.76±0.044	*e1-fs*	0.26±0.04	6.36±0.64

Plants were grown in a climate chamber under either SDs (12 h:12 h light/dark) or LDs (16∶8 h light/dark). Fully expanded trifoliolate leaves were sampled 4 h after beginning of light phase from three individual plants. Relative expression level of *GmFT4* and *E1* were analyzed by real-time RT-PCR. Transcript levels relative to *TUA5* are represented in each treatment, s.d. represents standard deviation. Soybean cultivar Kariyutaka under SDs was used as control whose expression level was set to 1 for all genes analyzed. Values represent means of three biological replicates. Genotype *E1* is considered as functional WT allele, the *e1-as* allele represents a partially functional allele and the *e1-fs* alleles are nonfunctional allele.

### Expression of *GmFT4* Exhibits a Diurnal Rhythm under LDs

**Figure 4:**
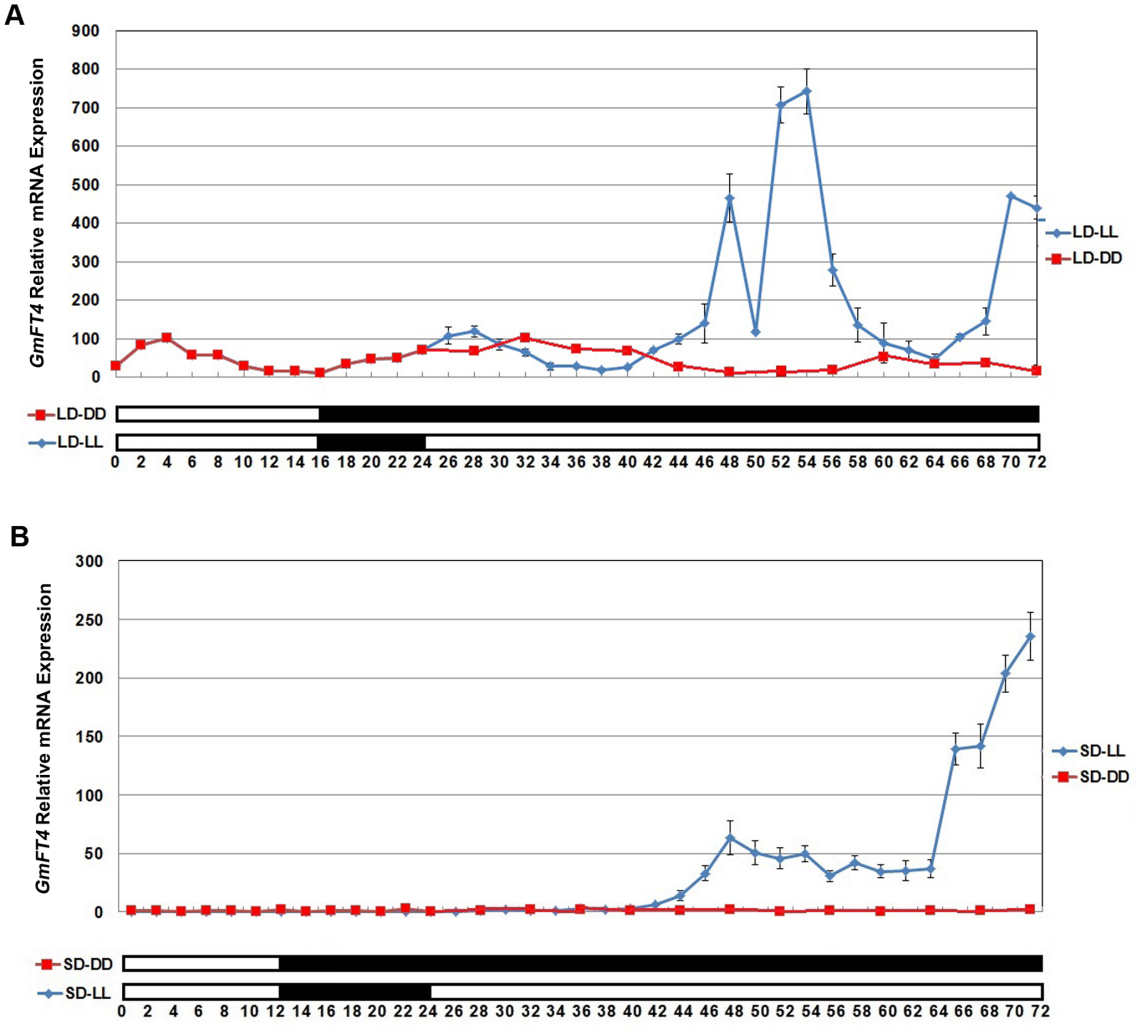
Diurnal expression pattern of *GmFT4* in Harosoy-*E1* fully expanded trifoliolate leaves. (**A**) *GmFT4* expression pattern in plants grown under long days (18∶6 h light/dark regime) followed by 48 h of continuous dark (LD-DD) or continuous light (LD-LL). (**B**) *GmFT4* expression pattern in plants grown under short days (12∶12 h light/dark regime) followed by 48 h of continuous dark (SD-DD) or continuous light (SD-LL). Transcript levels relative to *TUA5* are represented in each treatment; 2 h after beginning of the light phase under SD were used as control. Values represent means of three biological replicates; error bars indicate standard deviation. Leaves were sampled every 2 h under long days, short days and continuous light, every 4 h under continuous dark.

The diurnal rhythm of *GmFT4* gene expression was analyzed by real-time PCR in trifoliolate leaves sampled from cultivar Harosoy-*E1*. In plants under LDs, the expression level of *GmFT4* exhibits a diurnal rhythm. Briefly, the expression level increased beginning at dawn, reached a maximum 4 h later, then began to decrease and reached its minimum at the end of the light phase. In the dark phase, expression level of *GmFT4* began to increase again until 4 h after dawn in the next light/dark circle (Figure 4A).

When plants grown under LDs were transferred to subsequent 48 h continuous dark (LD-DD) condition, the oscillation waveform was similar to that under LDs, but expression peak of *GmFT4* appeared 4 h later than that under LDs in the first subjective cycle, and the expression peak drifted later by 4 h further in the second subjective cycle of darkness (Figure 4A). When plants grown under LDs were transferred to subsequent 48 h continuous light (LD-LL) condition, the expression waveform of *GmFT4* kept a similar pattern during the first subjective cycle of continuous light, but there was a sharp increase at the end of the first subjective cycle (Figure 4A). During the second subjective cycle of continuous light, basal expression level of *GmFT4* was generally elevated, but the rhythm became somewhat irregular.

In plants exposed to SDs, expression of *GmFT4* was very low and irregular (Figure S1). However, transcript level of *GmFT4* was increased after the shift to continuous light (SD-LL) (Figure 4B). The expression of *GmFT4* kept rising during the first 24 h of continuous light, and there was a large and sharp increase at the end of subjective cycle (Figure 4B). During the next subjective cycle of continuous light, the high expression level of *GmFT4* was maintained for a period, and then similarly there was a sharp increase at the end. By contrast, expression of *GmFT4* was kept at a very low level under continuous dark following SDs (SD-DD) (Figure 4B) and showed no circadian rhythm (Figure S1). The results indicated that the maintenance of expression rhythm of *GmFT4* needs a light/dark cycle, and a light phase can elevate the basal expression level.

### *GmFT4* Expressed Primarily in Leaves

**Figure 5:**
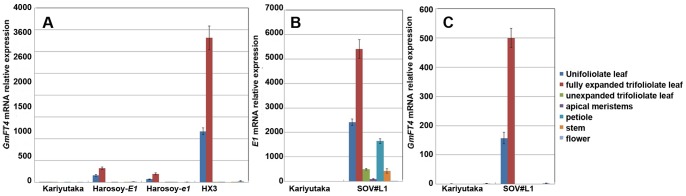
Tissue-organ expression analysis of *GmFT4* in different soybean cultivars. All tissues were sampled 2-time RT-PCR analysis. Transcript levels relative to *TUA5* are represented in each treatment. Values represent means of three biological replicates; error bars indicate standard deviation. (**A**) Tissue-specific organ expression analysis of *GmFT4* in soybean cultivars Kariyutaka, Harosoy-*E1*, Harosoy-*e1* and HX3. (**B**) Tissue-organ expression analysis of *E1* in *E1* overexpressing transgenic soybean and WT (Kariyutaka). (**C**) Tissue-organ expression analysis of *GmFT4* in *E1* overexpressing transgenic soybean and WT (Kariyutaka).

We analyzed transcription profiles of *GmFT4* in various tissues in near-isogenic lines Harosoy-*E1* and Harosoy-*e1*, cultivars Kariyutaka and HX3 (carrying *J* locus) under LDs by real-time PCR (Figure 5). The expression of *E1* was generally tissue-specific, with high levels in mature leaves (fully expanded unifoliolate leaves and trifoliolate leaves), relatively low expression levels in flowers, and very low but nearly identical levels in apical meristems, petioles and stems (Figure 5A). The expression levels of *GmFT4* in fully expanded unifoliolate leaves, trifoliolate leaves and flowers in the late flowering cultivar HX3 were higher than that in the corresponding tissues of the early flowering cultivar Kariyutaka. Also higher expression level of *GmFT4* in fully expanded unifoliolate leaves, fully expanded trifoliolate leaves and flowers was observed in Harosoy-*E1* than that in Harosoy-*e1*.

We further concurrently analyzed the expression level of *E1* and *GmFT4* in various tissues in *E1* overexpression transgenic soybean plants and wild type (Kariyutaka). As shown in Figure 5B, although an extremely high expression level of *E1* was observed in all tissues analyzed in transgenic soybean overexpressing *E1*, *GmFT4* was only highly induced in leaves (including fully expanded unifoliolate leaves and trifoliolate leaves) and slightly induced in flowers of the transgenic soybean (Figure 5C), implying that *GmFT4* may function primarily in leaves, and the induction of *GmFT4* by *E1* is conditioned by other genetic or molecular factors that are primarily present in leaves.

### Ectopic Expression of *GmFT4* in *Arabidopsis* Delayed Flowering

**Figure 6:**
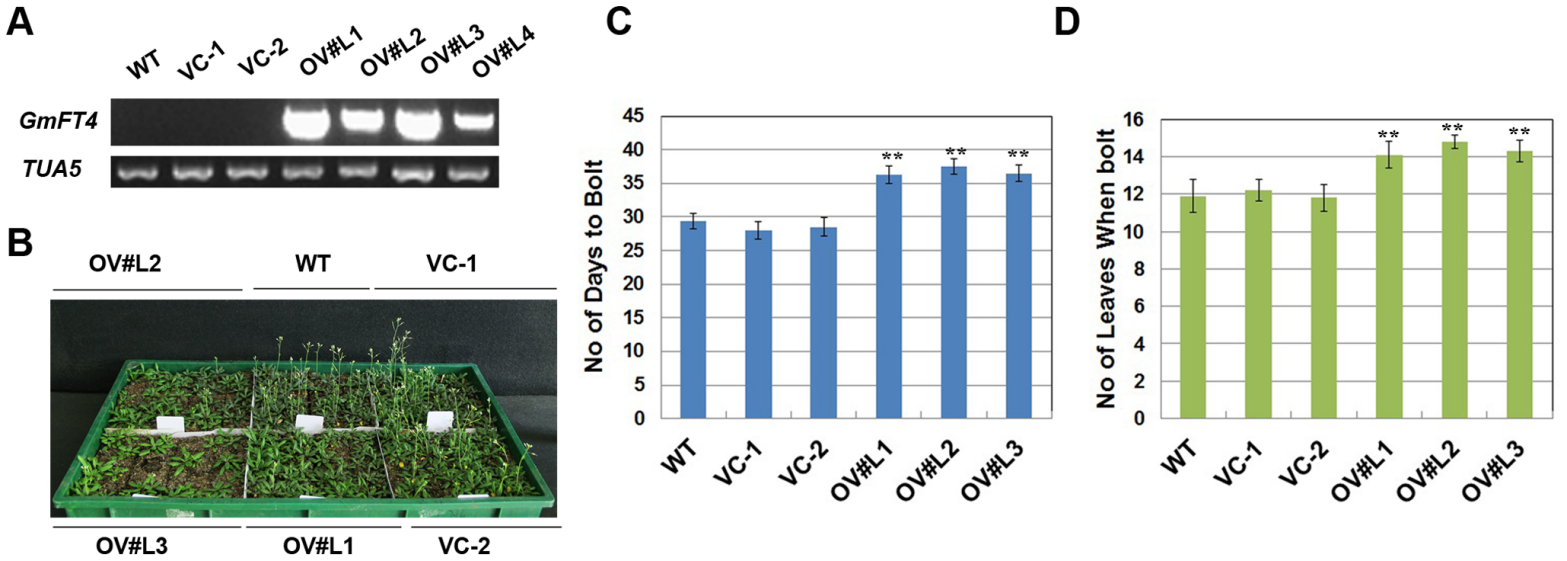
Effect of ectopic expression analyses of *GmFT4* in *Arabidopsis*. (**A**) Semi-quantitative RT-PCR analysis of transcript levels of *GmFT4* in four 35 S: *GmFT4* overexpression lines (OV#L1, OV#L2, OV#L3 and OV#L4). (**B**) Appearance of the indicated genotype plants 30 days after sowing under LD condition. (**C**) Flowering time measurement of the indicated plants. The time at which the main inflorescence shoot had elongated to 1 cm was recorded. (**D**)The number of rosette leaves when plants were flowering. Ninety plants were measured and averaged for each measurement and statistical analysis.

In order to further understand the function of *GmFT4* in flowering regulation, we conducted an ectopic overexpression experiment in *Arabidopsis* ecotype Columbia (Col-0). T_3_ homozygous lines were obtained and were confirmed by semi-quantitative RT-PCR with gene-specific primers. As shown in Figure 6A, four transgenic *Arabidopsis* lines all exhibited high expression levels of *GmFT4*, and no expression was detected in wild-type or vc (transformation vector only) transgenic plants.

Transgenic *Arabidopsis* with overexpression of *GmFT4* flowered later than the wild-type and vc plants under LDs (Figure 6B). Wild-type *Arabidopsis* and vc plants needed only 27–29 days to flower on average, while *GmFT4* overexpression lines needed 36–37 days to flower under LDs(Figure 6C). When flowering, wild-type *Arabidopsis* and vc plants had 11–12 rosette leaves on average, while *GmFT4* overexpression lines had 14–15 rosette leaves on average (Figure 6D). Obviously, the phenotype of transgenic *Arabidopsis* overexpressing *GmFT4* was distinct from that of other soybean flowering promoting *FT* homologs, *GmFT2a/5a* [21].

## Discussion

Each soybean cultivar is agronomically adapted to a narrow range of latitude for their maximal yield due to photoperiod sensitivity, thus limiting widely distribution of elite soybean cultivars. Among the major maturity genes or QTLs that have been reported so far, the *E1* gene has the most prominent effect on flowering time and photoperiod sensitivity in soybean [48], [60]–[62], suggesting *E1* is a key regulator of flowering in soybean. Phylogenetic analysis on protein sequence indicated *E1* is legume-specific [37], implying that the major photoperiodic pathway in soybean might be different from that in *Arabidopsis* and rice. Negative correlation between *E1* and *GmFT2a/GmFT5a* expression was observed in our previous study [37]. Here, we found that a *FT* ortholog, *GmFT4*, was positively associated with *E1* in *E1* overexpressing transgenic soybean (Figure 1), *E1* NILs and different soybean cultivars (Figure 3B). Interestingly, unlike most *FT*-like genes, *GmFT4* was characterized as a flowering repressor (Figure 6). These results indicated soybean has evolved a different strategy to regulate flowering time, and the *E1*-*GmFT4* pathway is valuable for understanding the molecular mechanisms of flowering time regulation in soybean.

*GmFT4* transcript level was strongly induced in *E1* overexpressing transgenic soybean lines (Figure 1), and Harosoy-*E1* showed a greater *GmFT4* transcriptional abundance than Harosoy-*e1* (Figure 3B). Allelic variations and transcriptional abundances of *E1* prominently influence *GmFT4* expression (Table 1). In addition, the expression pattern of *GmFT4* under SDs and LDs was similar to that of *E1* with a strong suppression under SDs and strong elevated expression under LDs (Figure 3B). *GmFT4* and *E1* have similar functions where both act as flowering repressors (Figure 6). All the results in this study indicate that *GmFT4* is regulated by *E1,* and *GmFT4* might be involved in the *E1* mediated flowering control pathway. However, *GmFT4* might be not the direct target of *E1*, since *E1* acts as a transcriptional repressor (data not shown).

In plants, the regulation of *FTs* in different species is highly diversified. Most *FT*-like genes are flowering activators and their induction occurs only in daylength that induce flowering. Not surprisingly, high *GmFT4* expression level in non-inductive LDs is consistent with its flowering repressing function. In *Arabidopsis* and rice, response to photoperiod is mediated by transcriptional regulation of *FTs* through an intersection between clock regulation and daylength. The expression of *FT* requires the activation of the clock output gene *CO* in the presence of light in *Arabidopsis* [10]. *FT* transcription is activated by *CO* under LDs but not under SDs, because under LDs, *CO* mRNA expression coincides with exposure of plants to light leading to stabilization of CO protein [9]–[10]. Here, we found that induction of *GmFT4* occurred in non-inductive daylength, and the diurnal oscillation expression pattern could be retained to some extent when transferred to subsequent 24 h continuous light or continuous dark, indicating that *GmFT4* was partly impacted by the circadian clock, and moreover that the induction of *GmFT4* required the exposure of plants to light, which indicated, just like other *FT*-like genes, transcriptional regulation of *GmFT4* also through an intersection between clock regulation and daylength.

Although *GmFT4* is preferably induced under LDs while *GmFT2a/5a* is preferably induced under SDs, the oscillation waveforms of *GmFT4* and *GmFT2a/5a* under respectively inductive conditions were similar, with an increase at the beginning of dawn, a peak 4 h later, and a minimum toward dusk, and then followed by an increase again, suggesting that these genes might be regulated by a similar mechanism in relation to the circadian clock.

In plants, *FTs* are highly conserved in different species. *Arabidopsis* has been used for functional confirmation of genes cloned, especially for *FT* homologs from different species. *ZCN8* encodes a FT-related protein in a SD plant maize. Ectopic expression of *ZCN8* accelerated flowering in transgenic *Arabidopsis* [23]. An antagonistic pair of *FT* homologs, *BvFT1* and *BvFT2* controls flowering time in LD plant sugar beet. Transgenic expression of *BvFT2* in both *Arabidopsis* and sugar beet strongly promoted flowering, while transgenic expression of *BvFT1* strongly repressed flowering in both *Arabidopsis* and sugar beet [29]. Previous studies indicated that the functions of *FT* genes are conserved between *Arabidopsis* and soybean. Ectopic overexpression of *GmFT2a* and *GmFT5a* in *Arabidopsis* showed a flowering promoting phenotype [21]–[22]. Conversely, when *Arabidopsis FT* was transformed into soybean, transgenic soybean flowered earlier [63]. Accordingly, we used *Arabidopsis* to confirm the function of *GmFT4*. When *GmFT4* was overexpressed in *Arabidopsis*, transgenic *Arabidopsis* showed a delayed flowering phenotype (Figure 6).

GmFT4 was grouped within the FT-like clade and carries functionally important FT signatures, but acts as a flowering repressor. Upon detailed analysis by referring to the previous publications, we predicted that the residue in position 143 that lies in the external loop may be critical for function diversification. Most FT-like proteins carry Gly(G) or Glu(E) residue in this position, except for flowering repressors GmFT4 and BvFT1 from *Beta vulgaris.* However, further evidence at molecular level is needed to verify this hypothesis.

**Figure 7:**
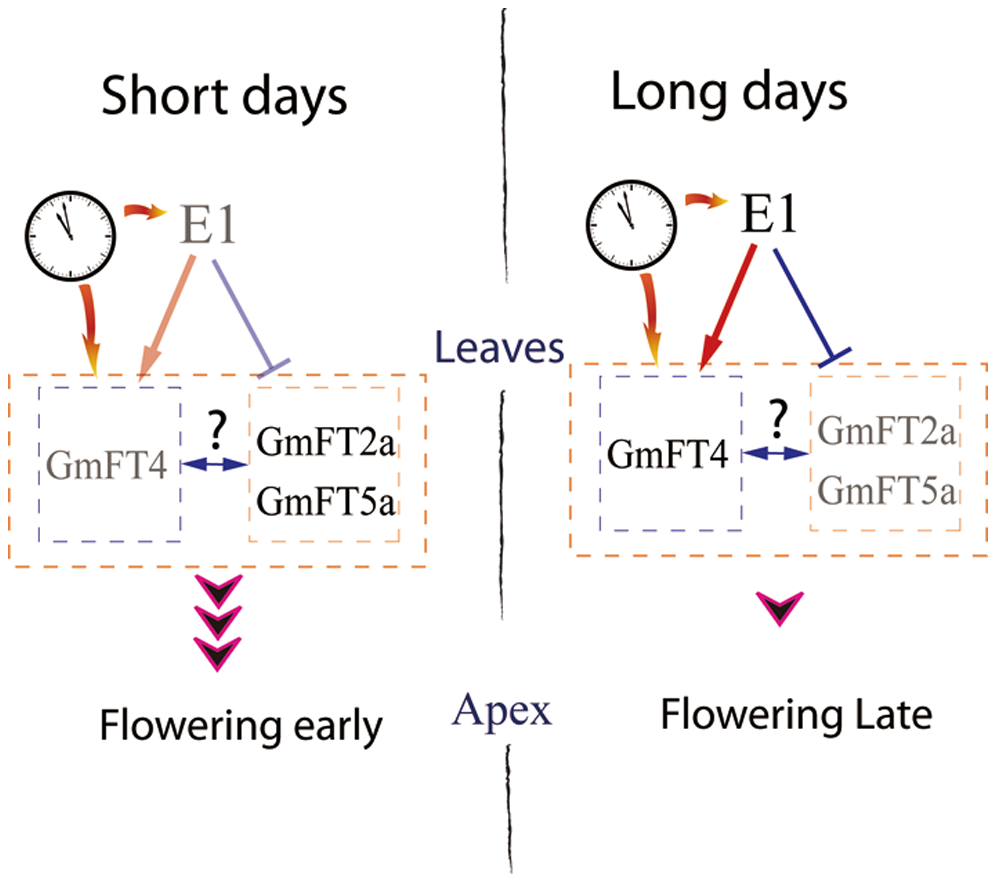
A proposed *E1-GmFT4/GmFT2a/GmFT5a* regulation model in soybean. (Some components and their positions were referred to Figure 7 of Xia et al [37].) Flowering repressor *GmFT4*, and flowering promoters *GmFT2a*/*GmFT5a* are all downstream of *E1*. *E1* positively regulates *GmFT4* transcription, but negatively regulates *GmFT2a/5a* transcription. The balance between the antagonistic FTs decides soybean flowering time. Arrows represent stimulation of the gene expression, T-shaped symbol represent inhibition of gene expression; The grayed out parts represent strength decline.

Most soybean cultivars have a short-day requirement for floral induction, so under long days, flowering and maturing are delayed and differ greatly among different cultivars. Here, we found the transcriptional abundance of *GmFT4* is significantly correlated with the flowering time of different soybean cultivars under LDs, indicating that *GmFT4* might be related to the flowering time regulation under LDs. Soybean cultivars grown at high latitudes are often photoperiod insensitive, because soybean plants planted in spring are required to flower under LDs during early summer and complete seed production in the limited frost-free season. Photoperiod insensitive cultivars grown at high latitude, such as Kariyutaka, Heinong 48, MuFeng 7 and Sakamotowase displayed low levels of *GmFT4* expression. Even under SDs, transcriptional abundance of *GmFT4* was also significantly correlated with the flowering time of different soybean cultivars. At low latitudes, soybean cultivars with the classic response to photoperiod flower early resulting in short plants and low grain yield [64], so soybean cultivars bred at low latitudes often needs the long juvenile period trait that featured as delayed flowering under SDs [65]–[67]. In this study, cultivar HX3, known for the Brazilian long juvenile period trait exhibited a delayed flowering phenotype under SDs. Generally, cultivars with the long juvenile period trait need a SD regime of 8 h/16 h (light/dark) to promote flowering. Here, we found cultivar HX3 also showed a relatively high *GmFT4* expression even under SDs, indicating that *GmFT4* might be an important determinant for flowering time regardless of day-length conditions. We assume that *GmFT4* might contribute greatly to the wide adaptability of soybean to wide range of latitudes. Hence, we proposed a model for the flowering time regulation in soybean (Figure 7). In this model, all flowering promoters *GmFT2a* and *GmFT5a,* and repressor *GmFT4*, function downstream of *E1*, and the balance between the antagonistic *FTs* (*GmFT4* vs *GmFT2a/GmFT5a* ) determines soybean flowering time. Under SDs or in cultivars carrying nonfunctional *E1* alleles or devoid of *E1* expression, the expression of the flowering repressor *GmFT4* is very low except for cultivars carrying long juvenile trait, and the expression of flowering promoters *GmFT2a/5a* are high, leading to an initiation of the flowering process. Under LDs, the expression of flowering repressor *GmFT4* is high, while flowering promoters *GmFT2a/5a* are repressed, so the flowering process is delayed.

Taken together, we conclude that *GmFT4* is positively regulated by *E1* and functions as a flowering repressor in soybean. Considering that soybean transformation is particularly difficult and time-consuming, we did not employ a soybean transformation approach for functional confirmation. In the future, characterization of *GmFT4* in soybean using various approaches including transgenic soybean will be needed to confirm the function of *GmFT4*. Future examination of the functional mechanism of GmFT4 will determine whether the GmFT4 protein can move like other florigens from leaves to the shoot apical meristem and will shed light on the precise regulation of photoperiodic flowering pathway in soybean.

## Supporting Information

Figure S1 Diurnal expression pattern of *GmFT4* in plants grown in SDs followed by continuous dark. 2 h after beginning of the light phase under SD were used as control. Values represent means of three biological replicates; error bars indicate standard deviation. Fully expanded trifoliolate leaves from Harosoy-*E1* were sampled every 2 h under short days, and every 4 h under continuous dark.
(DOC)
