# Supplementary material for: GmFT4, a Homolog of FLOWERING LOCUS T, Is Positively Regulated by E1 and Functions as a Flowering Repressor in Soybean
Source: PLoS One. 2014 Feb 19;9(2):e89030. doi: 10.1371/journal.pone.0089030 (PMC3929636; doi:10.1371/journal.pone.0089030)
Supplement: Figure S1 — Diurnal expression pattern of GmFT4 in plants grown in SDs followed by continuous dark. 2 h after beginning of the light phase under SD were used as control. Values represent means of three biological replicates; error bars indicate standard deviation. Fully expanded trifoliolate leaves from Harosoy-E1 were sampled every 2 h under short days, and every 4 h under continuous dark. (DOC) [file pone.0089030.s001.doc]

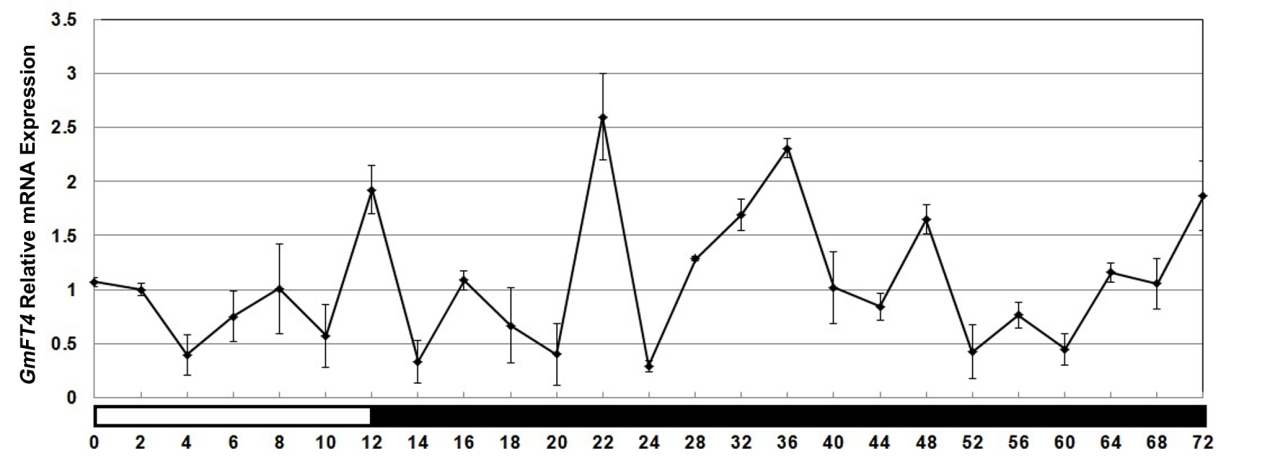


**Figure S1. Diurnal expression pattern of *GmFT4* in plants grown in SDs followed by continuous dark.** 2h after beginning of the light phase under SD were used as control. Values represent means of three biological replicates; error bars indicate standard deviation. Fully expanded trifoliolate leaves from Harosoy-*E1* were sampled every 2h under short days, and every 4h under continuous dark.
